# Tuning in to How Neurons Distinguish between Stimuli

**DOI:** 10.1371/journal.pbio.0040118

**Published:** 2006-03-21

**Authors:** Mason Inman

Most anyone can tell grape juice from fine wine with a quick sip, but distinguishing a French Syrah from an Australian Shiraz requires a more refined palette, plus perhaps a bout of sniffing and swirling. Similarly, our sensory neurons’ ability to make distinctions depends on the task at hand: in some cases, they excel at making coarse distinctions; in others, they are better at hairsplitting.

Finding out what exactly particular neurons are responding to is not as easy as asking a fellow diner which wine they prefer. But using a mathematical measure of information, a new study by Daniel Butts of Harvard University and Mark Goldman of Wellesley College reveals when specific cells are best at discriminating among different stimuli.

Listening in on neuronal activity, neuroscientists can see that a field of vertical stripes flashed in front of a cat’s eyes, for example, will goad a certain set of neurons into firing frenetically. Similarly, horizontal stripes will set another set of neurons firing, and diagonal stripes will activate all these neurons at some intermediate level.

So tweaking the stimuli—say, rotating the stripes—shows how a particular neuron or set of neurons responds to various stimuli, and the set of results can be represented with a so-called tuning curve. These curves are typically bell-shaped with a peak around one point where neurons are firing most quickly—corresponding, say, to stripes held vertically—and drop off to low activity on either side.

However, it hasn’t been clear which part of the tuning curve is most significant: does the peak reflect the stimuli a neuron is best at detecting? Or are the steep slopes of the curve most important, since this is where a small change in the stimulus elicits the greatest change in the neuron’s response? Studies of actual and simulated neuronal activity have given mixed results, without an overarching theory to explain them all.

In the new study, Butts and Goldman applied a mathematical measure of information, which they call the stimulus-specific information (SSI), to neurons’ tuning curves. If a neuron has a very reliable response to a certain stimulus, it is straightforward to spot this response. But if the neuron fires more sporadically, it is harder to say for sure if the neuron is responding to that stimulus or not. By calculating the SSI for neurons’ responses, Butts and Goldman were able to quantify how this uncertainty affected the ability of a neuron to convey information about various stimuli.

The analysis reveals that this noise in the neurons’ response is crucial to figuring out when neurons are most discriminating. In general, when the neurons’ responses were less noisy, the neurons were best at fine discrimination. In this case, a graph of the SSI could resemble a silhouette of Batman’s head: two sharp peaks, corresponding to the slopes of the tuning curve, with a smaller bump in the middle, matching the tuning curve’s peak. When the neurons’ response was noisy, however, the neurons were making more coarse distinction, and the graph of the SSI mirrored the tuning curve, showing a single peak where the neuron was firing most rapidly.

With their measure of SSI, Butts and Goldman have shown a way to connect neurons’ responses with how they process information and signal their neighbors. So just because a neuron is firing lackadaisically, that doesn’t mean it’s not getting the job done.

## 

**Figure:**
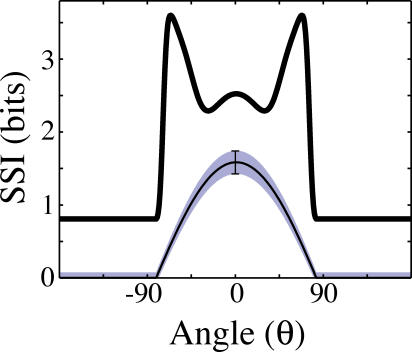
This “Batman” graph shows which stimuli are best encoded by a neuron in a relatively noise-free situation, when the neuron is better at making fine distinctions.

